# Sex Differences in the Renal Vascular Responses of AT_1_ and Mas Receptors in Two-Kidney-One-Clip Hypertension

**DOI:** 10.1155/2021/8820646

**Published:** 2021-02-20

**Authors:** Zahra Pezeshki, Mehdi Nematbakhsh

**Affiliations:** ^1^Water and Electrolytes Research Center, Isfahan University of Medical Sciences, Isfahan, Iran; ^2^Department of Physiology, Isfahan University of Medical Sciences, Isfahan, Iran; ^3^Isfahan^MN^ Institute of Basic and Applied Sciences Research, Isfahan, Iran

## Abstract

**Background:**

The prevalence and severity of hypertension, as well as the activity of the systemic and local renin angiotensin systems (RASs), are gender related, with more symptoms in males than in females. However, the underlying mechanisms are not well understood. In this study, we investigated sex differences in renal vascular responses to angiotensin II (Ang II) administration with and without Ang II type 1 and Mas receptor (AT_1_R and MasR) antagonists (losartan and A779) in the 2K1C rat model of renovascular hypertension.

**Methods:**

Male and female 2K1C rats were divided into 8 experimental groups (4 of each sex) treated with vehicle, losartan, A779, or A779+losartan. Responses of mean arterial pressure (MAP), renal blood flow (RBF), and renal vascular resistance (RVR) to Ang II were determined.

**Results:**

In both sexes, the basal MAP, RBF, and RVR were not significantly different between the four groups during the control period. The Ang II administration decreased RBF and increased RVR in a dose-related manner in both sexes pretreated with vehicle or A779 (*P*_dose_ < 0.0001), but in vehicle pretreated groups, RBF and RVR responses were different between male and female rats (*P*_group_ < 0.05). AT_1_R blockade increased RBF and decreased RVR responses to Ang II, and no difference between the sexes was detected. Coblockades of AT_1_R and MasR receptors increased RBF response to Ang II significantly in males alone but not in females (*P*_group_=0.04).

**Conclusion:**

The impact of Ang II on RBF and RVR responses seems to be gender related with a greater effect on males, and this sex difference abolishes by Mas receptor blockade. However, the paradoxical role of dual losartan and A779 may provide the different receptor interaction in RAS between male and female rats.

## 1. Introduction

Hypertension is a complex condition which induces abnormalities such as left ventricular hypertrophy, carotid atherosclerosis, and renal dysfunction [1–3], and it is the leading modifiable risk factor for death [2, 4]. Males have a higher prevalence of hypertension compared to age-matched premenopausal females [5, 6]. Hypertensive nephropathy also is gender dependent [7, 8]. Females are relatively resistant to renal and cardiovascular abnormalities than males. This has been attributed in part to physiologic sex-based differences in the function of the renin angiotensin system (RAS) [6, 9–11]. Despite these apparent gender differences in human hypertension, similar treatment guidelines are applied in both males and females [8].

Activation of systemic and local RAS via angiotensin II (Ang II) is an important contributing mechanism in multiple forms of hypertension [12–14]. Ang II type 1 receptor antagonists, as well as angiotensin-converting enzyme (ACE) and renin inhibitors, are widely used for treatment of hypertension [12–15]. Therapeutic strategies targeting the RAS have differential effects, in males and females, on the complications of hypertension, including renal disease [12, 14, 16, 17]. The mechanisms that underlie these differences are incompletely defined.

To improve management of hypertension, a better understanding of sex and gender differences in the function of the RAS, and in particular the renal responses to Ang II, is essential. In females, Ang II type 2 receptor (AT_2_R) and Mas receptor (MasR) are more abundant, while Ang II type 1 receptors (AT_1_R) are less abundant, in females than males [10, 11, 18]. Ang II has low affinity for the MasR, but interactions between the MasR and AT_1_R may alter the Ang II activity [19, 20]. However, these interactions have not been well documented in renovascular hypertension. In the current study, we tested the hypothesis that the relative roles of renal AT_1_R and MasR differ in males and females in two-kidney-one-clip (2K1C) renin-dependent hypertension. To test this hypothesis, we examined the effects of blockade of the AT_1_R (with losartan) and the MasR (with A779) on the renal response to Ang II in male and female rats with 2K1C hypertension.

## 2. Materials and Methods

### 2.1. Animals

The experiments were conducted on 64 (30 males and 34 females) in total age-matched male and female Wistar rats. All animals were housed in cages under controlled conditions (the room temperature of 23–25°C with 12 h light/12 h dark cycle) and had free access to water and rat chow. The experimental procedures were approved by the Ethics Committee of Isfahan Medical Sciences University (ethical code #: IR.MUI.REC.1397.3.461).

### 2.2. Surgical Procedures and Measurements

#### 2.2.1. Induction of 2K1C Hypertension

Fifty- to sixty-day-old male and female rats were anesthetized with chloral hydrate (450 mg/kg, i.p.) (Merck, Germany) and xylaxine (10 mg/kg, i.p.). The animals were subjected to right kidney clipping with a U-shaped silver clip (0.2 mm internal diameter). The right abdominal wall was shaved, and the skin and tissue of the right flank incised to expose the right kidney. The renal artery of the right kidney was isolated, and a clip was placed close to the aorta. Then, the kidney was carefully pushed back into the retroperitoneal cavity and the abdominal wall and skin were sutured. Povidone-iodine 10% was used for skin disinfection before and after surgery. Following the surgery, all rats were monitored until recovery and then individually housed for 24 h in a recovery cage and allowed access to standard rat chow and water ad libitum for 28 days post clipping.

#### 2.2.2. Sham-Operated Rats

We also assigned a total of 15 rats (7 males and 8 females) as sham-operated groups which were subjected to surgical operation process without renal clipping. The data from these sham groups were used to compare the blood pressures from 2K1C groups to verify hypertension in 2K1C rats.

#### 2.2.3. Catheterization and Measurements

Twenty-eight days after clipping, the rats (male: 276 ± 6, female: 184 ± 3 g) were anesthetized with urethane (1.7 g/kg i.p., Merck, Germany). Initially, the animals were tracheostomized to facilitate spontaneous ventilation. Then, the left carotid artery was catheterized for measurement of systolic (SYS) and mean arterial pressure (MAP). In addition, the left jugular vein was isolated, and the polyethylene catheter was implanted into the vein for drug infusion. Also, the left femoral artery was catheterized to determine renal perfusion pressure (RPP). The arterial pressures were measured by pressure transducers and bridge amplifiers connected to the PowerLab system (AD Instruments, Australia). An incision was made in the subabdominal area to insert a polyethylene catheter in the bladder to facilitate urine flow. Then, with the animals lying on the right side on a flat table, an incision was made in left flank using an electrical surgical cutter and the left kidney was carefully excised. To measure RBF, the renal artery was isolated followed by placing an ultrasonic flow probe (0.7 mm in diameter) (Transonic MA0.7PSB, flow probe, USA) directly around the renal artery. The probe was connected to a flow meter (T402, Transonic Systems Inc., Ithaca, NY 14850 USA). An adjustable occluder also was placed around the aorta between the renal and the mesenteric arteries to regulate RPP during Ang II infusion. MAP, RPP, and RBF were recorded continuously during the experiment. After surgical procedure, the animals were monitored for about 30 minutes to achieve the steady state condition. At the end of this period, the animal's SYS, MAP, RPP, RBF, and RVR (calculated by RPP/RBF ratio) were initially recorded as baseline data (control phase).

#### 2.2.4. Experimental Protocol

*(1) Vehicle/Antagonist Infusion*. Male rats receive saline (the vehicle group, group 1), AT_1_R blocker; losartan (the losartan group, group 2), MasR antagonist; A779 (Bachem Bioscience Inc., King of Prussia, PA, USA) (the A779 group, group 3); and the combination of A779 and losartan (the A779+losartan group, group 4). The female rats also had the same protocol as male rats of groups 1–4. Losartan and A779 were dissolved in 0.9% w/v saline. Losartan and A779 either alone or together were administrated in bolus doses of 5 mg·kg^−1^ and 50 *µ*g·kg^−1^, respectively, in relative groups, which then were followed by continuous infusions of 5 mg·kg^−1^·h^−1^ losartan and 50 *μ*g·kg^−1^ h^−1^ A779 with a microsyringe infusion pump (New Era Pump System Inc., Farmingdale, NY, USA) during the experiment. Vehicle-treated groups followed a similar protocol but received the saline vehicle instead of antagonists during the experiment. Subsequently, 30 minutes after vehicle/antagonist infusion, the parameters were measured and considered as data to determine the vehicle/antagonist effects (antagonist phase). However, the vehicle or antagonists were continuously infused until end Ang II administration (end of experiment).

**Figure 1:**
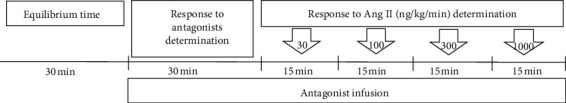
The protocol of experimental study (see Section 2.2.4).

*(2) Ang II Infusion*. The vascular responses to graded Ang II (Sigma, St Louis MI, USA) infusion were measured in all experimental groups, while the antagonists or vehicle injection continued during the Ang II infusion. Each dose of Ang II (0, 100, 300, or 1000 ng/kg/min) was administrated for 15 min, and the last 3-minute measurements in each dose were obtained. During Ang II administration, the aortic diameter was adjusted by an aortic occluder to control RPP at constant level. Finally, the rats were killed humanly, and the right and left kidney were rapidly removed and weighed. The protocol of the experimental study is demonstrated in (Figure 1)

#### 2.2.5. Statistical Analysis

Data are expressed as mean ± standard error of mean (SEM) and were analyzed using SPSS software (version 20). The baseline data were compared using the one-way analysis of variance (ANOVA) followed by the least significant difference (LSD) test. The paired-sample *T*-test was applied to compare the left and right kidney weights. The responses to antagonists or vehicle are reported as percentage (%) change from the baseline values prior to administration of the antagonists. Responses to Ang II are reported as % change from the average values after administration of the antagonists or its vehicle, but prior to administration of Ang II. These data were then subjected to ANOVA for repeated measures, with the between-subject factor “group” (*P*_group_), the within-subject factor “time” (*P*_time_), and their interaction (*P*_time&group_). *P* ≤ 0.05 was considered statistically significant.

## 3. Results

### 3.1. Verification of 2K1C Implementation

**Figure 2:**
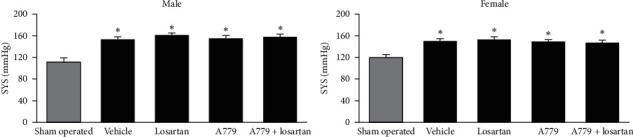
Systolic pressure (SYS, mmHg) in sham-operated and 2K1C groups. The data are reported as mean ± standard error of mean. ^*∗*^Significant difference from sham-operated groups. The statistical *P* values were obtained using one-way ANOVA followed by the LSD post hoc test.

The verification of 2K1C hypertension was determined on the day of experiment after direct blood pressure measurement. Two exclusion criteria were assigned to verify hypertension by 2K1C. The animals with SYS blood pressure lower than 145 mmHg and the rats with infarcted (partially or totally) kidney tissue were excluded from the study. Accordingly, the success rate of the accepted 2K1C hypertensive model was 32% in male and 31% in female rats. Therefore, a total of 64 (30 males and 34 females) 2K1C rats were included in this study. The SYS pressure in male 2K1C rats was 157 ± 2.2 mmHg and in female 2K1C rats was 148.8 ± 2.1 mmHg which was significantly different *P*=0.023. These data revealed less elevation of SYS pressure in female 2K1C rats compared with male 2K1C rats. The SYS blood pressure in male and female 2K1C groups was also compared with the sham-operated group (Figure 2). Significant differences were detected between 2K1C and sham-operated groups (*P* < 0.05). The comparison between right and left kidney weight per 100 gr body weight indicated that the weight of the clipped kidney (right kidney) was lower than that of an unclipped one in both sexes, while no significant difference between right and left kidney weight was observed in male and female sham-operated groups (Table 1). These data confirmed the induction of hypertension in the clip cased hypertension.

### 3.2. Baseline Data (Control Phase)

The baseline data within each sex showed that there was no significant difference in MAP, RPP, RBF, and RVR between the groups prior to administration of antagonists or vehicle (Table 2).

### 3.3. Hemodynamics Responses to Antagonist Infusion (Antagonist Phase)

**Figure 3:**
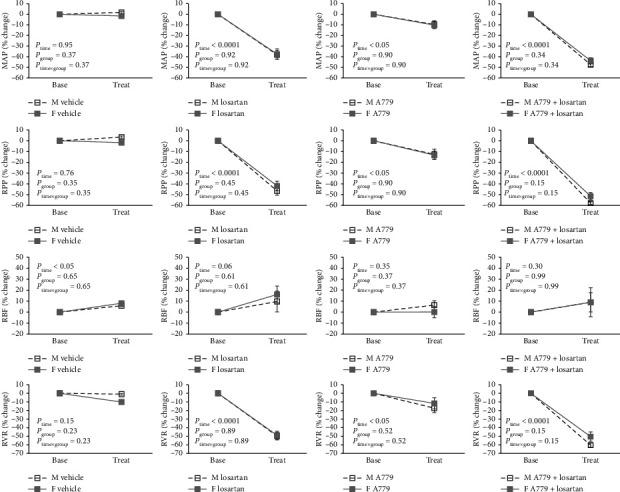
The effect of antagonist on hemodynamics parameters. MAP, RPP, RBF, and RVR stand for mean arterial pressure, renal perfusion pressure, renal blood flow, and renal vascular resistance in male M and female F rats. Data are presented as mean ± SEM and were analyzed using repeated-measures ANOVA with the factors group (sex), treat (antagonist), and their interaction.

The results of the antagonist phase indicated the reduction of MAP, RPP, and RVR percentage change in male and female rats treated with losartan alone and A779 plus losartan about 40–60 percent from the baseline. These findings confirmed the important role of Ang II action through AT_1_R in maintaining arterial pressure and causing vasoconstriction. On the other hand, A779 had little effect, and it does not appear to be important in basal control of hemodynamics parameters. However, no significant difference was detected between male and female rats in MAP, RPP, RBF, and RVR percentage change by vehicle or antagonists (Figure 3).

### 3.4. Hemodynamics Response to Ang II Administration

**Figure 4:**
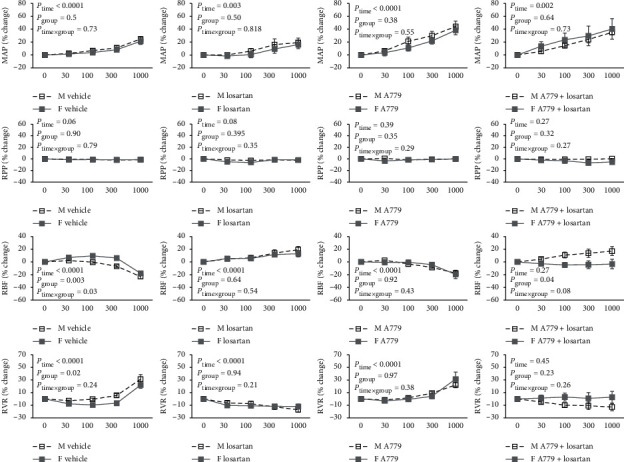
The effect of graded Ang II infusion on hemodynamics parameters. MAP, RPP, RBF, and RVR stand for mean arterial pressure, renal perfusion pressure, renal blood flow, and renal vascular resistance in male M and female F rats. Data are presented as mean ± SEM and were analyzed using repeated-measures ANOVA with the factors group (sex), time (dose of Ang II), and their interaction.

Ang II infusion increased MAP percentage change in male and female groups, and no differences were detected between the sexes (Figure 4). However, the raw values of MAP in the equilibrium time (base), 30 min after antagonist administration (treat), and graded angiotensin II infusion are tabulated in Table 3. As previously described, RPP was fixed by aortic occluder during Ang II infusion. So, no difference was expected in RPP percentage change response to Ang II between the male and female groups. Administration of losartan alone increased RBF percentage change response to Ang II infusion in male and female rats. For example, Ang II with dose of 1000 ng/kg/min increased RBF about 19.3% in male and 13% in female rats, while coadministration of A779 and losartan increased RBF response to Ang II (1000 ng/kg/min) 17.2% in male rats and decreased 3.3% in female rats (Figure 4). The Ang II infusion decreased RBF and increased RVR percentage change in a dose-related manner in both sexes, and these responses were significantly different between male and female rats (Figure 4). That means the greater RBF and RVR responses to Ang II were detected in male rats received vehicle instead antagonist when compared with female rats. However, this sex difference in RBF and RVR responses to Ang II was eliminated by AT_1_R and MasR antagonists' infusion. Administration of losartan alone decreased RVR response to Ang II infusion in male and female rats. For example, Ang II with a dose of 1000 ng/kg/min decreased RVR 17% in male and 12% in female rats, while coadministration of A779 + losartan decreased RVR response to Ang II (1000 ng/kg/min) 12.8% in male rats and increased 2.7% in female rats (Figure 4).

## 4. Discussion

Our major findings showed that in vehicle-treated rats, the renal vasoconstriction was greater in males than in females, confirming sex differences in the renal response to Ang II. Losartan abolished the renal vasoconstriction in response to Ang II, and in both sexes, renal vasodilatation was seen with no sex difference. Sex differences in the renal vasoconstrictor response to Ang II also were not seen after MasR blockade. This provides some level of evidence to support a role of MasR in underlying the sex difference in the response to Ang II. The most striking sex difference in the response to Ang II was after combined blockade of AT_1_R and MasR. Such finding highlights the role of gender and receptor interaction between male and female rats in the 2K1C model.

Our data support the fewer RBF and higher RVR responses to Ang II in female rats. It seems that these findings are related to hypertension and sex which affect RAS components. The RAS components such as renin, ACE, angiotensinogen, AT_1_R, AT_2_R, MasR, and AT_4_R are expressed in the kidney [14, 21–23], and different distributions of receptors in the kidneys and other organs have been observed in both sexes [24]. Hypertension itself changes distribution and regulation of vascular and renal RAS component which may be caused different systemic and renal responses [23, 25–27]. In the current model of hypertension, 2K1C-induced Ang II-dependent hypertension, it may change the systemic and local renal RAS distribution and function [28–30]. Ang II-dependent hypertension was gender related in kidney transplantations subjects [31]. Renal ACE2 mRNA expression level in males is less than that in females, and treatment with Ang II has enhanced ACE2 mRNA expression in females 2.5 folds greater than that in males [32]. 2K1C hypertension reduced vascular and renal AT_1_R [26, 27]. Also, a lower ratio of AT_1_R/AT_2_R is found in female rats than in male rats spontaneously hypertensive rats (SHRs) [18]. Ang II infusion decreased AT_1_R and increased AT_2_R in the renal cortex of male SHRs without change in AT_1_R and AT_2_R expression in female SHRs [33]. The enhancement of ACE/Ang II/AT_1_R pathways is observed in males, and the balance is shifted towards the ACE2/Ang 1–7/MasR and AT_2_R pathways in females [11, 24, 34]. Augmentation of the vasodilatory arm of the RAS in females contributes to the gender differences in response to Ang II.

AT_1_R antagonist, losartan, eliminated the vasoconstriction response to Ang II. The renal AT_1_R distribution and function in males is more than that in females [31]. Thus, the disappearance of the sex difference in vasoconstriction response to Ang II may be due to the elimination of the greater AT_1_R response to Ang II in male rats.

The other finding indicates that no differences were detected in RBF and RVR responses to Ang II infusion between males and females in condition of MasR blockade male rats have a greater blood pressure response to Ang II than female rats and A779 eliminated gender difference response to Ang II [33]. Coadministration of A779 and losartan in female rats had shown more RBF reduction compared to male rats. MasR participates in vasorelaxation and blood pressure reduction via Ang 1–7 [22, 35]. MasR is not activated by Ang II but indirectly involved in Ang II action through an interaction with the AT_1_R. Coexpression of MasR and AT_1_R inhibits the actions of Ang II and acts as a physiological antagonist for AT_1_R [19]. MasR distribution and function is also gender dependent with a greater distribution and function in female rats than in male rats [11, 36, 37]. In addition, a study indicated the enhancement of MasR protein expression in carotid of 2K1C hypertensive rats [35], while the renal Mas mRNA was not different between SHRs and normotensive Wistar rats [38]. Ang II infusion enhances MasR protein expression in female SHRs with no change in male SHRs [33]. Also there was not gender difference in the intratubular MasR expression 3 weeks after 2K1C while 5-week flowing clipping operation intratubular MasR expression was elevated in female rats compared with male 2K1C rats [39], and renal MasR was decreased in male 2K1C rats [40]. Ang 1–7 via MasR and Ang II via AT_1_R interact with each other on rat renal mesangial cells [41]. Women plasma Ang 1–7 is higher compared with men [42]. Renal cortical Ang 1–7 levels are higher in female SHRs compared with male SHRs [33]. Therefore, MasR blocking in the A779 groups eliminated vasodilatory effect of MasR and may remove the inhibitory effect of the MasR on AT_1_R and induce a greater response, especially in females, which have a greater distribution of the MasR.

## 5. Conclusion

The fewer RBF and RVR responses to Ang II in female rats may be related to more ACE2/Ang 1–7/MasR and AT_2_R pathway activities in female rats than in male 2K1C rats. Also, enhancement of Ang II due to 2K1C may affect distribution of different receptors and transporters in male and female rats and change the expected systemic and local RAS function. Moreover, the stronger RBF response to Ang II in A779 and A779 + losartan 2K1C female treated groups is related to greater distribution and function of ACE2/Ang 1–7/MasR arm of the RAS and fewer activity of ACE/Ang II/AT_1_R arm of the RAS in female 2K1C rats. Therefore, rather than blocking the ACE/Ang II/AT_1_R axis, activating the ACE2/Ang 1–7/MasR axis of the RAS may be more effective in improving the renal and systemic side effects of Ang II-dependent hypertension in female rats but not in male rats.

## Figures and Tables

**Table 1:** The male and female data for body weight before clipping operation (BW1) and 28 days after 2K1C implementation (BW2).

	Groups	BW1 (g)	BW2 (g)	*P*	RKW (g)/100 gBW	LKW (g)/100 gBW	*P*
Male	Sham operated (*n* = 7)	193 ± 7.2	277 ± 5.8	0 < 0.01	0.37 ± 0.03	0.36 ± 0.01	0.64
	Vehicle (*n* = 9)	192 ± 7.1	272 ± 7.3	0 < 0.0001	0.37 ± 0.01	0.47 ± 0.02	0 < 0.001
	Losartan (*n* = 8)	214 ± 9.2	280 ± 14.5	0 < 0.0001	0.29 ± 0.03	0.46 ± 0.04	0.05
	A779 (*n* = 7)	212 ± 7.1	256 ± 12.9	0.01	0.28 ± 0.04	0.48 ± 0.03	0.02
	A779 + losartan (*n* = 6)	225 ± 8.7	298 ± 7.1	0 < 0.0001	0.31 ± 0.01	0.36 ± 0.02	0.02

Female	Sham operated (*n* = 8)	151 ± 3.6	195 ± 5.9	0 < 0.0001	0.37 ± 0.02	0.35 ± 0.01	0.37
	Vehicle (*n* = 10)	149 ± 2.1	191 ± 3.7	0 < 0.0001	0.32 ± 0.02	0.42 ± 0.02	0.02
	Losartan (*n* = 7)	152 ± 2.7	182 ± 3.5	0 < 0.0001	0.36 ± 0.02	0.43 ± 0.02	0.05
	A779 (*n* = 9)	145 ± 7.5	187 ± 7.1	0 < 0.0001	0.33 ± 0.01	0.39 ± 0.01	0.02
	A779 + losartan (*n* = 8)	143 ± 2.2	173 ± 3.4	0 < 0.0001	0.27 ± 0.03	0.42 ± 0.03	0.03

The right kidney weight per 100 gram BW (RKWg/100 gBW) and left kidney weight per100 gram BW (LKWg/100 gBW). The data are reported as mean ± standard error of mean. The statistical *P* values were obtained using the paired-sample *T*-test.

**Table 2:** The baseline hemodynamics data.

	Groups	MAP (mmHg)	RPP (mmHg)	RBF (ml/min)	RVR (mmHg·min/ml)
Male	Vehicle (*n* = 9)	120.4 ± 6.4	109.1 ± 10.6	2.9 ± 0.2	41.9 ± 7.5
	Losartan (*n* = 8)	124.2 ± 3.3	115.4 ± 3.5	3.1 ± 0.3	41.2 ± 5.1
	A779 (*n* = 7)	112.7 ± 6.6	100.1 ± 7.3	2.6 ± 0.3	41.8 ± 5.3
	A779 + losartan (*n* = 6)	124.3 ± 6.8	114.1 ± 8.97	2.2 ± 0.3	57.3 ± 9.9
	P_(ANOVA)_	0.51	0.60	0.22	0.39

Female	Vehicle (*n* = 10)	114.8 ± 6.4	105.2 ± 5.5	2.4 ± 0.3	48.5 ± 6.1
	Losartan (*n* = 7)	116.2 ± 8.6	108.9 ± 9.3	2.3 ± 0.3	47.1 ± 5.5
	A779 (*n* = 9)	114.5 ± 3.5	108.0 ± 3.6	2.4 ± 0.3	50.0 ± 5.5
	A779 + losartan (*n* = 8)	113.2 ± 6.6	101.6 ± 6.6	1.8 ± 0.1	59.7 ± 5.8
	*P*_(ANOVA)_	0.99	0.85	0.35	0.48

MAP, RPP, RBF, and RVR stand for mean arterial pressure, renal perfusion pressure, renal blood flow, and renal vascular resistance, respectively. The data were reported as mean ± standard error of mean. The statistical *P* values were obtained using one-way ANOVA followed by the LSD post hoc test.

**Table 3:** Mean arterial pressure (MAP) in the equilibrium time (base), 30 min after antagonist administration (treat), and graded angiotensin II infusion.

	Mean arterial pressure (mmHg)						
	Antagonists			Angiotensin II (ng/kg/min)			
Treatment	Group gender	Base	Treat	30	100	300	1000

Vehicle	Male	120 ± 6.4	121 ± 5.0	124 ± 4.5	129 ± 4.8	134 ± 5.6	150 ± 5.2
	Female	114 ± 6.3	112 ± 4.8	113 ± 4.5	116 ± 4.3	120 ± 4.0	135 ± 4.5
		*P*_time_=0.8	*P*_group_=0.3		*P*_time_ < 0.0001	*P*_group_=0.04	
		*P*_time&group_=0.4			*P*_time&group_=0.5		

Losartan	Male	124 ± 3.3	76 ± 4.7	77 ± 5.4	81 ± 6.3	87 ± 6.5	91 ± 8.2
	Female	116 ± 8.6	72 ± 5.7	71 ± 6.4	72 ± 7.4	79 ± 8.9	83 ± 7.0
		*P*_time_ < 0.0001	*P*_group_=0.3		*P*_time_=0.004	*P*_group_=0.4	
		*P*_time&group_=0.7			*P*_time&group_=0.9		

A779	Male	113 ± 6.6	102 ± 8.1	108 ± 7.7	122 ± 7.3	131 ± 7.1	144 ± 6.3
	Female	114 ± 3.5	102 ± 5.4	106 ± 6.1	113 ± 6.0	124 ± 4.4	140 ± 5.1
		*P*_time_=0.001	*P*_group_=0.9		*P*_time_ < 0.0001	*P*_group_=0.5	
		*P*_time&group_=0.8			*P*_time&group_=0.5		

A779 + losartan	Male	124 ± 6.8	65 ± 2.5	68 ± 2.8	74 ± 3.5	81 ± 5.2	87 ± 3.5
	Female	113 ± 6.6	64 ± 6.0	70 ± 4.0	76 ± 4.9	78 ± 6.3	85 ± 5.8
		*P*_time_ < 0.0001	*P*_group_=0.5		*P*_time_ < 0.0001	*P*_group_=0.9	
		*P*_time&group_=0.1			*P*_time&group_=0.5		

Data are presented as mean ± SEM and were analyzed using repeated-measures ANOVA with the factors group (sex), time (treated with antagonist or dose of Ang II), and their interaction.

## Data Availability

The data used to support the findings of this study are available from the corresponding author on request.
